# Exploring Risk Factors and Sex Differences in Colorectal Cancer: Insights from Current Evidence

**DOI:** 10.3390/cells15111039

**Published:** 2026-06-05

**Authors:** Camilla Cittadini, Elisabetta Iessi, Rosa Vona, Paola Matarrese

**Affiliations:** 1 Department of Infectious Diseases, Italian National Institute of Health, Viale Regina Elena, 299-00161 Rome, Italy; camilla.cittadini@iss.it; 2 Center for Gender-Specific Medicine, Italian National Institute of Health, Viale Regina Elena, 299-00161 Rome, Italy; elisabetta.iessi@iss.it (E.I.); paola.matarrese@iss.it (P.M.)

**Keywords:** colorectal cancer, sex/gender differences, sex hormones, gut microbiome, precision oncology

## Abstract

Colorectal cancer (CRC) is the third most diagnosed malignancy and the second leading cause of cancer-related mortality worldwide. A consistent and epidemiologically well-documented feature of CRC is its sexual dimorphism: age-standardized incidence rates are 33–45% higher in men than in women, and mortality rates differ by 43–50%. Beyond epidemiology, biological sex influences tumor location, molecular subtype, and clinical outcome. Women more frequently develop right-sided, microsatellite-unstable tumors driven by the CpG island methylator phenotype pathway, whereas men predominantly present with left-sided, chromosomally unstable tumors harboring APC, KRAS, and TP53 mutations. Sex steroid hormones play a central modulatory role: estrogens, primarily via estrogen receptor β (ERβ), exert tumor-suppressive effects on colonic epithelium, whereas androgens promote pro-inflammatory and pro-tumorigenic signaling through androgen receptor (AR)-dependent pathways. The gut microbiome displays sex-specific compositional profiles (‘microgenderome’) and contributes to sex-specific CRC susceptibility through bidirectional interactions with sex hormones, shaping distinct immunological and metabolic microenvironments. Finally, sex influences the pharmacokinetics of fluoropyrimidines, the toxicity of targeted agents, and the response to immune checkpoint inhibitors. This review summarizes current evidence on sex-related differences in CRC epidemiology, molecular pathology, hormonal regulation, gut microbiota composition, and treatment outcomes, highlighting the need to systematically incorporate sex as a biological variable in CRC research and clinical practice.

## 1. Introduction

Colorectal cancer (CRC) is a significant global health concern, being the third most diagnosed cancer and the second leading cause of cancer-related deaths worldwide [1,2]. It is the third most common cancer in men and the second most common in women. Despite advances in screening and treatment, the incidence of CRC has increased in recent years, with projections estimating over 2.2 million new cases and 1.1 million CRC-related deaths per year by 2030 [1,3]. Specifically, the rate of CRC has been increasing quickly in emerging nations, while stabilizing or falling trends have been seen in advanced countries, where levels stay high [4]. A salient feature of CRC epidemiology is its pronounced sexual dimorphism [2,5,6]. Its incidence and mortality rates are consistently higher in men than in women, and this disparity persists across various geographical regions and age groups [2,7]. While historical perspectives primarily attributed these differences to behavioral factors, such as dietary habits, smoking and alcohol consumption, contemporary research has shifted towards intrinsic biological determinants, including sex steroid hormones, genetic susceptibility and gut microbiome composition [2,7].

Several studies have highlighted the existence of sexual dimorphism in CRC development, and interest in examining the potential crosstalk between sex, sex hormones and gender in colon oncogenesis has grown in recent years [2,5,6]. A thorough understanding of the mechanisms underlying the observed sex- and gender-related differences in CRC could facilitate the development of innovative, more effective and personalized therapeutic approaches.

This review aims to provide a comprehensive overview of CRC epidemiology and clinical characteristics, with a particular focus on the roles of sex hormones, gut microbiota, and CRC therapeutic treatments.

## 2. Sex Difference in the Epidemiology, Clinical Features, and Molecular Characteristics of CRC

### 2.1. Epidemiology

CRC remains a paramount global health concern, currently ranked as the third most common malignancy and the second leading cause of cancer-related mortality worldwide, with approximately 1.9 million new cases and over 900,000 deaths annually [2,8]. Nearly 10% of CRC is classified as early-onset (EO) CRC, once it is diagnosed at a younger age (before the age of 50) [9,10]. All the other cases are known as late-onset (LO) CRC. Epidemiological data consistently highlight a significant male predominance in both incidence and mortality rates across nearly all geographical regions [2,7,11]. Global statistics indicate that the age-standardized incidence rate is 33–45% higher in men than in women, and the overall mortality rate is 43–50% higher in men [5,11]. Specifically, men have an incidence rate of around 23.4 to 23.6 per 100,000 person-years, compared to 16.2 to 16.3 for women [5,12]. This sexual dimorphism persists across various ethnicities and levels of socioeconomic development, although absolute rates vary markedly by country [5,13,14]. This reflects the combined influence of behavioral and biological factors. Men are more frequently exposed to risk factors, such as alcohol and tobacco consumption, and a diet rich in red and processed meats, and have a higher prevalence of visceral obesity. In contrast, women tend to have healthier eating habits and engage in more regular preventive behaviors [15,16]. Sex-related differences in hormone levels and physical activity are additional biological factors. Interestingly, when data for women is broken down by age, a trend known as the ‘female paradox’ emerges: women have a lower risk of colorectal cancer for most of their lives, but a higher incidence and mortality rate than men after the age of 65–70. This finding is supported by various data, which backs up the conclusions that were drawn [13,17,18]. This suggests that the protective advantage of the female sex may diminish post-menopause [13,18]. Furthermore, Kim (2026 highlights that, although overall CRC rates in older adults have stabilized in high-income countries due to effective screening, there has been an alarming increase in EOCRC among individuals under 50 years of age [19]. Survival patterns also exhibit sex-specific nuances: Rodriguez-Santiago et al. (2024) confirm that premenopausal women (aged 18–44) generally demonstrate better survival rates than age-matched men [18]. This is often attributed to the protective role of endogenous estrogens [2,20]. Thus, the interaction between biological, hormonal and behavioral factors influences not only the incidence of CRC in the two sexes, but also its clinical progression and survival outcomes [2,21].

Taken together, the global trends reported by Wong et al. (2021) emphasize that gender is a critical determinant of CRC risk that requires tailored public health strategies [22].

### 2.2. Sex Difference in the Clinical Characteristics of CRC

The clinical presentation of CRC is characterized by a distinct anatomical localization between the sexes. This phenomenon is widely referred to as ‘sidedness’ [5,12]. Extensive research confirms that women are significantly more likely to develop right-sided (proximal) colon cancer, whereas men more frequently present with left-sided (distal) tumors and rectal cancer [6]. Tsokkou et al. (2025) report that women with colon cancer have a right-sided tumor proportion of approximately 59.2%, compared to a higher prevalence of distal tumors in men [2]. This anatomical divergence has profound clinical implications: right-sided tumors, which originate from the embryonic midgut [23], often exhibit a more aggressive phenotype and are associated with poorer long-term outcomes. From a diagnostic perspective, right-sided lesions in women are often flat rather than polypoid, making them considerably more difficult to detect during standard colonoscopies than the more visible left-sided polyps prevalent in men [5]. The difficulty of visualizing these lesions, combined with the often vague or asymptomatic nature of proximal tumors, often results in diagnosis at more advanced stages, or even as emergency cases [17,23,24,25]. Symptomatology also varies by location: right-sided CRC is typically associated with iron-deficiency anemia due to chronic occult blood loss and abdominal pain, whereas left-sided CRC often presents with rectal bleeding and changes in bowel habits, such as diarrhea or narrow stools [23]. Screening tools such as the fecal immunochemical test (FIT) may be less sensitive in women because proximal tumors bleed less consistently, resulting in higher rates of interval cancers [2,12]. Recent evidence suggests that adopting sex-specific fecal hemoglobin (HbF) thresholds could address this diagnostic gap. Indeed, large-scale studies have demonstrated that women typically exhibit lower HbF concentrations than men, resulting in higher false-negative rates for advanced neoplasia at standard thresholds [26]. Furthermore, evidence from the Stockholm-Gotland screening program indicates that employing sex-specific cut-offs (e.g., 40 µg Hb/g for women versus 80 µg Hb/g for men) can balance positivity rates and improve the identification of advanced lesions in females [27]. Considering the ‘female paradox’ and the more aggressive behavior of right-sided colorectal lesions in postmenopausal women, adjusting the starting age or frequency of screening could help to reduce the higher mortality rate observed in older female populations [28,29], Although colorectal cancer typically develops four to five years later in women than in men, women have a higher prevalence of right-sided tumors, which are often more aggressive and clinically silent. Therefore, initiating more intensive screening strategies from the age of 45 could improve early detection before the postmenopausal increase in risk.

These findings reinforce the conclusion of Schmuck et al. (2020) that sex should be considered a key feature in personalized diagnostic protocols but, we add, also in gender-differentiated screening protocols [29].

### 2.3. Molecular Characteristics

**Figure 1:**
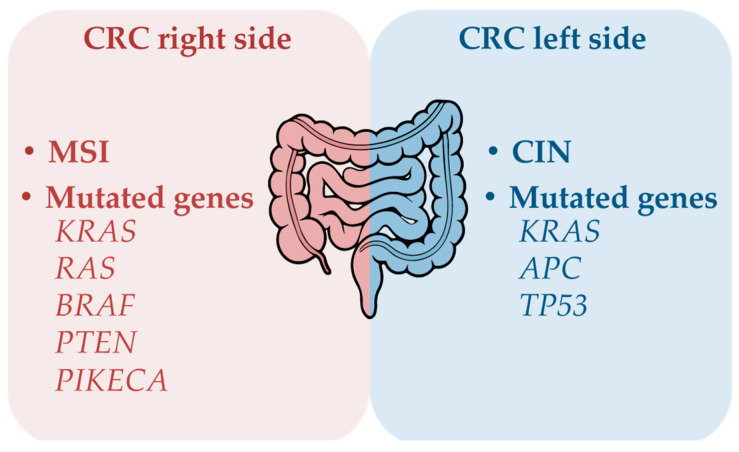
Molecular characteristics of left- and right-sided CRC. The main molecular characteristics are observed on the left and right sides of CRC. MSI, microsatellite instability; KRAS, Kirstern Rat Sarcoma viral oncogene homolog; RAS, Rat Sarcoma; BRAF, V-Raf murine sarcoma viral oncogene homolog B1; PTEN, Phosphatase and Tensin homolog; PIK3CA, Phosphatidylinositol-4,5-bisphosphate 3-kinase catalytic subunit alpha; CIN, chromosomal instability; APC, Adenomatous Polyposis Coli; TP53, Tumor Protein 53. Adapted from citation [2].

Colorectal cancer is a molecularly heterogeneous disease with distinct carcinogenic pathways that are strongly influenced by biological sex [5,30]. The majority of CRC tumors are sporadic (70–80%), whereas approximately 20% are hereditary [31]. The most mutated genes in CRC patients are APC (82%), TP53 (48–59%), KRAS (40–50%) and PIK3CA (14–18%) [32]. The mutational status of some genes is important in CRC carcinogenesis (e.g., NRAS, KRAS, HER2 and BRAF), or it is associated with defects in the DNA mismatch repair (MMR) system. A deficient MMR system leads to a hypermutable phenotype characterized by microsatellite instability (MSI), whereby the insertion or deletion of repeating units (microsatellites) are not corrected during DNA replication. Right-sided tumors, which are more prevalent in women, are frequently driven by the microsatellite instability (MSI-high) pathway, the CpG island methylator phenotype (CIMP) and BRAF mutations [5,13,30,33,34]. The rate of MSI tumors is significantly higher in females (22.9%) than in males (12.0%), which has profound implications for responsiveness to immunotherapy [2]. In contrast, left-sided tumors, which are more common in men, typically follow the chromosomal instability (CIN) pathway, which is characterized by mutations in the APC, KRAS and P53 genes [2,5] (see Figure 1).

**Figure 2:**
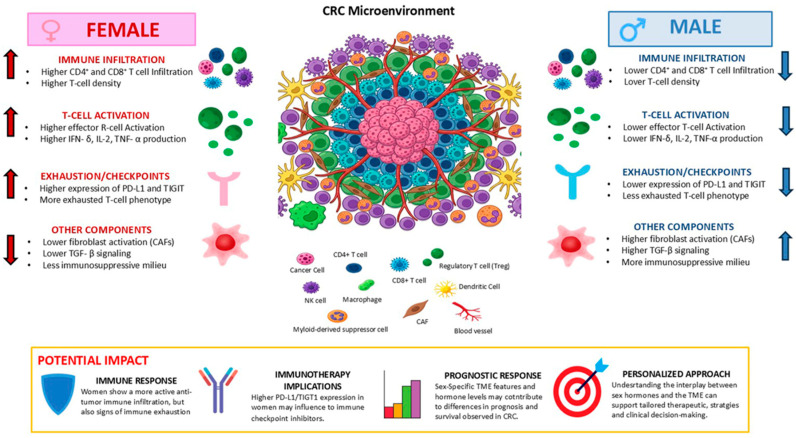
Sex-specific characterization of the tumor microenvironment (TME) in colorectal cancer (CRC). This figure illustrates the distinct differences in the TME between female ((**left**), pink panel) and male ((**right**), blue panel) patients. The diagram highlights a key paradox: women show a significantly higher immune infiltration (with greater CD4+ and CD8+ T-cell levels, T-cell density, and effector activation, indicated by ↑ arrows) compared to men, but simultaneously exhibit higher expression of immune checkpoint markers (PD-L1, TIGIT) and a more exhausted T-cell phenotype. Conversely, men display lower T-cell infiltration and activation (indicated by ↓ arrows), alongside higher levels of cancer-associated fibroblasts (CAFs) and TGF-β signaling (↑ arrows), contributing to a more immunosuppressive milieu. The ‘Potential Impact’ box summarizes how these gender-specific characteristics influence the anti-tumor immune response, the implications for immunotherapy, prognostic outcomes, and the development of personalized treatment strategies for patients with colorectal cancer. NK, Natural Killer cells; CAF, Cancer-Associated Fibroblasts; PD-L1, Programmed Death-Ligand 1; TIGIT, T cell immunoreceptor with Ig and ITIM domains; IFN-γ, Interferon-γ; TNF-α, Tumor Necrosis Factor-α.

The tumor microenvironment (TME) also reveals striking sexual dimorphism: women generally exhibit higher levels of CD4+ and CD8+ T cell infiltration, suggesting more robust baseline immune surveillance [2,12]. However, female tumors also exhibit elevated markers of immune exhaustion, such as PD-L1 and TIGIT, which may affect treatment response surveillance [2,12] (see Figure 2).

Estrogen plays a central role in these molecular differences, primarily through its interaction with the estrogen receptor beta (ERβ), which acts as a tumor suppressor and is ubiquitous in normal colonic mucosa [6,18]. The literature explains that ERβ activation promotes anti-proliferative signals and inhibits the Wnt/β-catenin signaling pathway, which is a major driver of cell proliferation [7]. In males, a lower activation threshold for Wnt signaling and the presence of tumorigenic genes on the Y chromosome promote more rapid cancer progression. Interestingly, most of the genes involved in the onset and progression of CRC differentially expressed in the two sexes are located on the X or Y chromosomes [35]. For example, genes such as KDM5C and ATRX, which are located in areas of the X chromosome that escape inactivation, may be overexpressed in females compared to males, providing women with greater genomic stability, whereas the expression of Y chromosome genes such as KDM5D has been linked to more aggressive phenotypes in men [2]. In a transcriptomic study using data from The Cancer Genome Atlas (TCGA), Hases et al. [30] proposed 20 genes with potential sex-specific prognostic biomarkers. Among these, ESM1, GUCA2A, and VWA2 in males and CLDN1 and FUT1 in females showed a more robust correlation with overall survival (OS).

Distinct methylation patterns of key genes, including MLH1 (MutL homolog 1 gene encodes a protein crucial for DNA MMR), and APC (Adenomatous Polyposis Coli, tumor suppressor gene controlling the Wnt signaling pathway), further suggest epigenetic differences between the sexes [36]. Epigenetic changes, such as the levels of p16INKα methylation, also account for gender-related differences in CRC onset. These changes are more prevalent in women than in men [37]. Differences in micro-RNA expression were also observed in male and female CRC patients. In particular, an experimental study involving CRC patients (72 males and 26 females) demonstrated that two miRs considered as possible biomarkers in CRC, miR-21 and miR-26, showed significantly higher expression levels in tumor tissues of men than in women, making these miRs potentially useful as gender-specific markers [38].

Overall, the interaction between oncogenic pathways, hormonal regulation, epigenetic architecture and the immune response contributes to molecular dimorphism in CRC, influencing incidence, progression and therapeutic response. This highlights the critical role of biological sex as a key variable in precision medicine.

## 3. Sex Differences and Risk Factors in CRC

**Figure 3:**
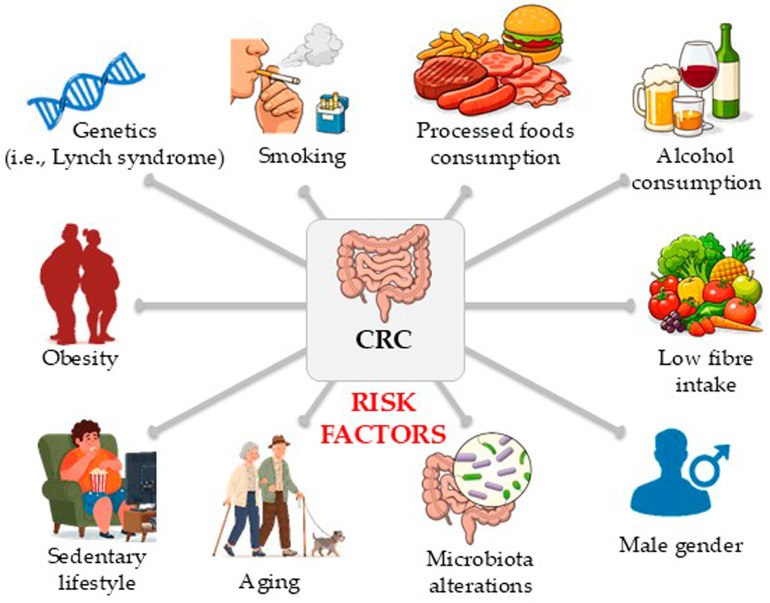
Risk factors for CRC. A schematic representation of factors associated with the development of colorectal cancer including modifiable factors (moderate to heavy alcohol consumption, smoking, a diet high in fat and low in vegetables, obesity, and sedentary lifestyle), non-modifiable factors (age, inflammatory bowel disease), and hereditary components.

The development of colorectal cancer is determined by a complex interaction between non-modifiable sex-related biological factors and modifiable gender-related behaviors [4,13,14]. In this review, biological sex and gender are considered as distinct but interacting dimensions. Biological sex encompasses the chromosomal, gonadal, hormonal and anatomical characteristics that distinguish males from females and directly influences carcinogenic processes through sex steroid signaling, immune regulation and gene expression. Gender, by contrast, refers to socially constructed roles and behaviors that modulate exposure to modifiable risk factors (dietary habits, alcohol and tobacco use, physical activity and participation in screening). Both dimensions contribute to the observed dimorphism in CRC: sex operates primarily through biological mechanisms, gender through behavioral and socio-economic pathways. Although the molecular mechanisms involved remain unclear, the development of CRC is strongly linked to modifiable factors such as lifestyle, alcohol consumption, and smoking habits. However, metabolic conditions currently considered pathological, such as obesity, also play a fundamental role (see Figure 3). Numerous lines of evidence support these associations, particularly regarding their influence on specific molecular subtypes of CRC [15,39]. Dietary habits further widen the gap, as women typically maintain healthier diets than males, consuming more fiber and vegetables and less meat and alcohol [5,12,13,40]. Further complicating the picture, lifestyles differ by sex in their impact on the development of CRC.

Evidence suggests that the impact of diet on CRC risk varies by sex and tumor location [41,42]. For instance, a high inflammatory profile, defined by a pro-inflammatory diet, sedentary behavior, and obesity, is strongly associated with CRC risk in males, but not in females. Additionally, high carbohydrate intake is associated with right-sided colon cancer in females and rectal cancer in males, while high fat and protein intake specifically increases the risk of right- and left-sided colon cancers [5]. Moreover, it has been proven that eating meat can increase the risk of left-sided colon cancer [43,44].

Excess weight is usually caused by an imbalance in the long term between the energy we take in and the energy we use up. Body weight can be strongly influenced by our eating and exercise habits. Obesity is a well-established risk factor, but its impact varies significantly between the sexes. Indeed, several studies report that a higher body mass index (BMI) is more strongly associated with an increased risk of CRC in men than in women [2,19]. For women, central obesity, measured by waist-to-hip ratio (WHR), appears to be a much stronger predictor of CRC risk than BMI alone [2,12,15]. This is partly because, in postmenopausal women, adipose tissue becomes a significant source of endogenous estrogen, which can exert a local protective effect against tumor initiation [5,13].

Tobacco smoking is another critical factor with sex-specific consequences. Several studies show that male smokers are at a higher risk of distal tumors, whereas female smokers are more predisposed to developing proximal and rectal cancers [2,45].

Alcohol consumption also exhibits sex-based thresholds. Jin et al. (2023) [46] proposed that women may be more susceptible to alcohol-induced CRC, even in small quantities, due to variations in metabolism and body composition [13]. It has been demonstrated that the ingestion of ethanol can cause localized irritation of the upper gastrointestinal tract and stimulate carcinogenesis by inhibiting DNA methylation [5].

Beyond individual behaviors, Martinez et al. use a social epidemiology approach to estimate that gender-related mechanisms, such as social role expectations and healthcare access, account for 30–50% of the association between sex assigned at birth and CRC incidence [14].

Finally, the sex hormone–gut microbiome axis is emerging as a novel risk modifier. Wu et al. (2024) state that estrogen promotes a more diverse and favorable gut microbiota, whereas androgens may favor opportunistic pathogens that exacerbate inflammation [7].

Understanding how these multiple risk factors intertwine and influence each other is mandatory for developing gender-sensitive prevention and intervention programs.

## 4. Environmental Pollutants and CRC

Exposure to environmental pollutants is a risk factor for all diseases, particularly non-communicable diseases, such as cardiovascular disease and cancer, including CRC. Environmental pollutants can affect men and women differently, interacting with both sex-related biological factors and behavioral factors related to social role, and therefore gender-dependent, which can manifest differently in different countries and/or social contexts. Environmental pollutants can contribute to the development of CRC indirectly by inducing chronic inflammation, oxidative stress, and alteration of the gut microbiota [47,48]. In men, who are not protected by estrogen, these effects may be more pronounced than in women of childbearing age. Addressing the broad topic of the role of environmental pollution in the development and progression of cancer, and CRC in particular, would require a dedicated paper, but even in the context of this review, we cannot fail to mention at least the main pollutants implicated as recognized risk factors for CRC. Air pollution has a primary impact, as expected, on respiratory health. However, the substances present in the air also profoundly affect intestinal health.

Long-term exposure to fine particulate (PM2.5) and nitrogen oxides (NO_2_) has been reported to significantly increase the incidence of and mortality from CRC, respectively [49,50]. The importance of PM2.5 as a risk factor in the development of CRC was recently highlighted by an American study which reported that for every 1 μg/m^3^ increase in PM2.5 concentration resulting from the combustion of fuel oil, the incidence of CRC increased by 15.6%, and for PM2.5 resulting from the combustion of coal, this increase was 7.3% [51].

It is no coincidence that living in industrial areas with high environmental risks exposes the population not only to increased incidence and mortality, but also to different tumor localization. For example, in areas with high levels of air pollution, there is an overall increase in CRC cases, but with a lower incidence in the rectal tract and a significantly higher incidence in the colon localization of the lesions [52]. On the other hand, living in rural areas exposes people to different types of environmental pollutants, such as pesticides, herbicides, antifungals, insecticides, etc. Analysis of literature data and a meta-analysis of recently published studies seem to indicate a clear association between exposure to herbicides and insecticides and colon cancer, both in relation to the intensity of exposure and its duration [53]. Although the mechanisms by which these chemicals induce or promote the development of CRC have not yet been sufficiently studied, the precautionary principle would suggest that policymakers and regulatory bodies adopt more restrictive regulations on the production and use of pesticides, in order to minimize the risk of colorectal cancer both in the most exposed population and in workers involved in their production and/or use.

Several pesticides are identified as endocrine disruptors, which interfere with the hormonal pathways and may increase the risk of hormone-sensitive cancers, including CRC [53].

Among pesticides, neonicotinoids such as acetamiprid, used mainly for the control of sucking insects on crops such as fruits, vegetables and ornamental plants, can easily penetrate plant tissues, leading to human exposure mainly through diet [54]. Recently, it has been highlighted that this class of pesticides induces oxidative stress in adipocytes, altering metabolism and contributing to the development of obesity [55]. Women, due to their greater abundance of adipose tissue, therefore, appear to be particularly vulnerable to the toxic effects of neonicotinoids, shifting the metabolic balance toward a more severe dysfunctional state [56].

Synthetic polymers such as polyvinyl chloride, polycarbonate, polypropylene, and acrylic can degrade over time, releasing nano (smaller than 1000 nm) and microplastics (smaller than 5 mm) into the environment. Numerous clinical and experimental studies indicate a positive correlation between nano- and microplastic particles and CRC. For instance, a significantly higher presence of microplastic particles has been detected in CRC tissues than in the corresponding healthy tissues [57]; furthermore, an increased risk of CRC has been observed in workers of plastic and rubber manufacturers [58].

It has been hypothesized that the increased incidence of colorectal cancer in individuals under the age of 50 has been contributed to, among other causes, by greater exposure to plastic, the use of which has become widespread since the 1950s and 1960s and which has led to a growing accumulation of micro- and nano-plastics in the environment [59]. Although the mechanisms through which microplastics promote the onset and progression of CRC are not yet fully elucidated, chronic inflammation, alteration of the intestinal barrier and the intestinal microbiota are believed to play a fundamental role. Microplastics can release bisphenol A (BPA) during their degradation, or they can act as carriers of BPA which binds to their surface due to its affinity for polymer surfaces.

BPA is a chemical compound used as a plasticizer in the production of polycarbonates and epoxy-phenolic resins. Present in various everyday consumer products, such as food contact materials and thermal paper, it inevitably meets the entire population. Under certain conditions, BPA is released into the environment and into food and can therefore be ingested upon contact with the intestines. BPA is one of the most studied endocrine disruptors due to its widespread use.

It binds to ERβ without triggering any receptor activity [60]. By inhibiting both the genomic and non-genomic activity of E2, BPA would neutralize the protective effects of E2 on CRC [61,62]. Women would therefore be at greater risk of developing CRC following BPA exposure.

Among environmental pollutants, heavy metals have recently been associated with CRC. A study by Yongsheng Li and colleagues highlighted significantly higher levels of vanadium (V), arsenic (As), tin (Sn), barium (Ba), lead (Pb), chromium (Cr), and copper (Cu) in CRC patients compared to the control group, while Cr, Cu, As, and Ba were identified as risk factors for CRC development. An interesting observation arising from this study is the positive association between antimony (Sb) and thallium (Tl) with BRAF V600E mutations, leading to MSI [63].

Although heavy metals induce biological effects in both sexes, the health risks appear to differ between men and women [64]. For example, for the same levels of occupational exposure to lead, higher blood levels are observed in women. The greater vulnerability to heavy metals observed in women has been associated with a higher percentage of body fat, a site of accumulation of many heavy metals.

Sex hormones can also play a dual role in this context. Estrogens, thanks to their antioxidant properties, can play a protective role against heavy metals, but thanks to their effect on metabolism and detoxification, they can also increase their absorption. On the other hand, testosterone has been reported to mitigate the effects of some heavy metals.

The susceptibility to heavy metal toxicity may vary by sex. Sex-related factors, such as disparities in the absorption, distribution, metabolism, and excretion of heavy metals, or gender-related factors, such as lifestyle and exposure, may be responsible for the differences in vulnerability to heavy metals observed between men and women.

Most research on the health impacts of environmental pollutants is designed to ignore potential gender differences. Similarly, meta-analyses of published data fail to stratify data by sex/gender, a significant limitation to knowledge in this field. In fact, it is important to consider that, in general, female biology interacts with pollutants in a more complex way than male biology, especially in relation to the different hormonal states in which women find themselves (for example, the different phases of the menstrual cycle, menopause and pregnancy). This requires a personalized approach to prevention, diagnosis, and treatment.

## 5. Sex Hormones and CRC

Sex hormones are an important modulator of CRC risk, involved in both development and progression, with significant differences in the effects observed between males and females. As mentioned in the Section 2.1, premenopausal women have a lower risk of CRC than men of the same age. Furthermore, hormone replacement therapy (HRT) has been associated with a reduced incidence of CRC in postmenopausal women [47]. It is therefore clear that estrogen plays a very important role in the onset of CRC. In addition to estrogen, studies have shown that androgens and progesterone also influence key tumorigenic processes by regulating proliferation, apoptosis, cell differentiation, and interactions with the intestinal microenvironment [22,48,49,50].

Sex steroid hormones are primarily produced in the gonads, but the necessary enzymes for their production have been found in other peripheral tissues, such as the colon. These hormones include 17β-estradiol, testosterone, and progesterone, and their respective receptors, estrogen receptor α and β (ERα and ERβ) and G protein-coupled estrogen receptor (GPER), progesterone receptor (PGR), and androgen receptor (AR, existing homo- or heterodimers), which are also expressed by the colonic mucosa [51,52,53]. Below is a brief discussion of these factors and their role in the onset of CRC.

### 5.1. Estrogens

Estrogens play an important role in regulating intestinal homeostasis and modulating processes involved in colorectal carcinogenesis. Of the various physiological forms, 17β-estradiol (E2) is the most biologically active estrogen in humans, followed by estrone (E1) and estriol (E3) [6]. These steroid hormones are derived from cholesterol via androgen intermediates through aromatization, a process catalyzed by the aromatase enzyme [6]. The main sites of synthesis are the gonads and adrenal glands, but local production in peripheral tissues also significantly contributes to regulating tissue concentrations [65,66]. There is a great deal of epidemiological and experimental evidence indicating that estrogens play a protective role in CRC. As previously mentioned, a reduction in estrogen levels after menopause is associated with an increased risk of CRC. Conditions involving estrogen deprivation, such as oophorectomy or hysterectomy, result in an increased risk between 24% and 30%. Conversely, estrogen therapy has been associated with favorable effects on intestinal physiology, suggesting direct action of estrogens on colorectal tissue [7].

The biological effects of estrogens are mediated by binding to specific estrogen receptors (ERs), which belong to the nuclear receptor superfamily. The two main isoforms, ERα and ERβ, are encoded by the ESR1 and ESR2 genes, respectively, and differ in their distribution and function [67,68]. Both receptors are located in the nucleus and the cytoplasm, as well as on plasma membranes and mitochondrial membranes [69]. In normal colon tissue, ERβ is the predominant isoform and plays a key role in maintaining epithelial integrity by promoting the expression of antiproliferative and pro-apoptotic genes [70,71,72].

**Figure 4:**
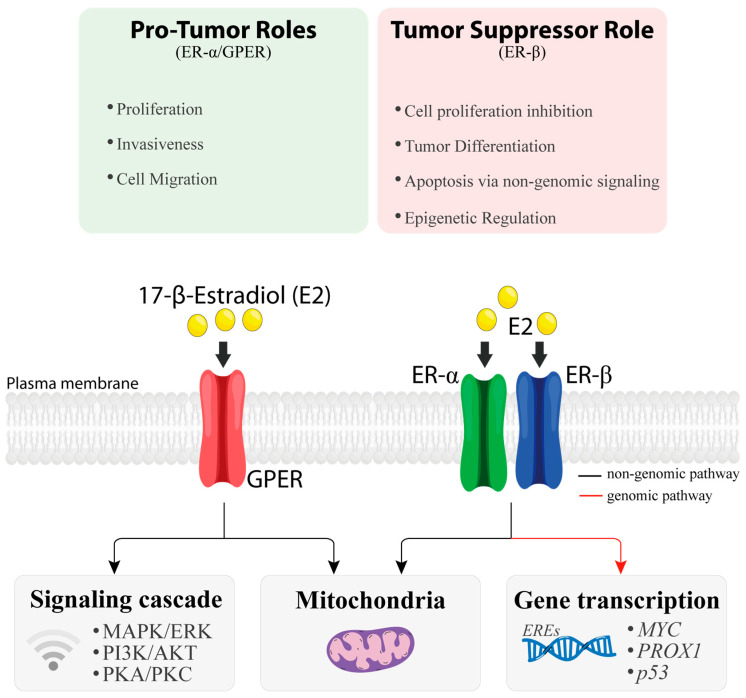
Mechanism of action of estrogen in CRC. Estrogen (E2) binds to estrogen receptors (ERα, ERβ and GPER), thereby modulating the expression and activity of multiple signaling pathways. In the genomic pathway (red arrow), E2 interacts with estrogen response elements (EREs) within target gene promoters or regulates gene transcription through interactions with other transcription factors. The black arrows show the E2-induced cellular and mitochondrial ER/GPER non-genomic effects, which regulate mitochondrial respiration, ATP production and ROS formation. The green and pink boxes illustrate the effects of the ERβ, and ERα and GPER activation in CRC, respec-tively. ERα, -β Estrogen Receptors; GPER, G- protein-coupled estrogen receptor 1; Prox1, Prospero Homeobox 1; p53, protein 53; NF-kB, Nuclear factor kappa-light-chain-enhancer of activated B cells; COX-2, Cyclooxygenase-2; NRF2, Nuclear Factor Erythroid 2-Related Factor 2; MAPK, Mitogen-Activated Protein Kinase; ERK, Extracellular signal-Regulated Kinase; PI3K, Phosphoinositide 3-kinase; AKT, Protein Kinase B. Adapted from citations [7,69,80].

From a molecular perspective, ERs have a modular structure consisting of a DNA-binding domain (DBD), a ligand-binding domain (LBD), and regulatory regions that allow modulation of transcriptional activity [67,73,74,75]. The mechanism of estrogen action can involve genomic or non-genomic signaling effects. After binding to estrogen, the receptors dimerize and translocate to the nucleus, where they regulate gene expression by binding to estrogen response elements (EREs), producing effects known as ‘genomic’ [67,76]. Additionally, estrogens can modulate genomic transcription via ERE-independent mechanisms by interacting with transcription factors such as AP-1 and Sp-1 [75,77] (see Figure 4).

Estrogens activate rapid, non-genomic signaling pathways via plasma membrane receptors (ERα and ERβ) and the membrane G protein-coupled receptor GPER [78,79]. Activation of these systems induces signal transduction through the MAPK/ERK, PI3K/AKT, PKA and PKC pathways, as well as the production of intracellular second messengers. These processes influence proliferation, survival, migration and the cellular stress response by modulation of reactive oxygen species (ROS) [76,80]. The integration of genomic and non-genomic signals constitutes a complex regulatory network that modulates cellular behavior within the tumor microenvironment [28,81] (see Figure 4).

During CRC progression, a progressive reduction in ERβ expression is observed. In some cases, this is accompanied by a relative increase in ERα, leading to an imbalance in favor of proliferative and survival signals [7,71]. ERβ is known to exert tumor-suppressive effects by negatively regulating oncogenes such as MYC and PROX1, enhancing DNA repair mechanisms, and activating p53-dependent pathways. Conversely, ERα promotes the activation of the PI3K/AKT and MAPK/ERK pathways, which are associated with tumor growth [7,28]. Interestingly, in tumors other than CRC, such as non-small cell lung cancer, the role of ERα and ERβ is completely reversed and it is the activation of the latter that has a protumor role through the activation of the MAPK/ERK, PI3K/AKT, PKA pathways [82,83].

The ERα/ERβ ratio, together with the contextual activation of GPER, which mediates rapid, non-genomic estrogen signaling, plays a crucial role in determining CRC progression. Literature data confirm that a predominance of ERβ is associated with less aggressive phenotypes, whereas a relative increase in ERα and, in specific contexts, GPER promotes proliferation, invasiveness, and disease progression [6,7,18,28,71,82,84,85]. Experimental studies support the anti-tumor role of E2 in CRC, highlighting a reduction in the proliferation and migration of neoplastic cells, the inhibition of inflammatory pathways mediated by NF-κB and cyclooxygenase-2 (COX-2), and the enhancement of antioxidant defenses through the activation of nuclear factor erythroid 2-related factor 2 (Nrf2). In animal models, E2 administration reduces polyp and tumor formation, whereas estrogen deprivation increases their incidence. These effects are also associated with strengthened cell junctions mediated by E-cadherin and β-catenin, creating a microenvironment that is less favorable to tumor progression [7,86].

Understanding the molecular mechanisms underlying this balance is crucial for explaining the differences in incidence and prognosis between the sexes and for developing preventive and therapeutic strategies that selectively modulate estrogen signaling.

### 5.2. Androgens

Testosterone (T) and dihydrotestosterone (DHT) are both androgens with DHT produced by the conversion of testosterone via the enzyme 5-alpha-reductase. Although typically considered male hormones, androgens also play an important role in female physiology. In males, they are primarily produced by the testes, with a minor contribution from the adrenal glands, whereas in females they are mainly synthesized by the ovaries and adrenal glands.

Androgens exert their biological effects through androgen receptors (ARs), which are widely expressed and regulate multiple physiological and proliferative processes [87]. Two main AR isoforms have been identified, AR-A and AR-B. Both isoforms are present in normal colonic mucosa, whereas only AR-A is detected in neoplastic tissue, indicating a loss of AR-B expression during malignant transformation [87,88]. In the colon, androgen signaling occurs via both nuclear androgen receptors (nARs), which regulate gene transcription, and membrane androgen receptors (mARs), which mediate rapid non-genomic responses. While AR expression is low in normal colonic tissue, it increases significantly during tumorigenesis [85].

**Figure 5:**
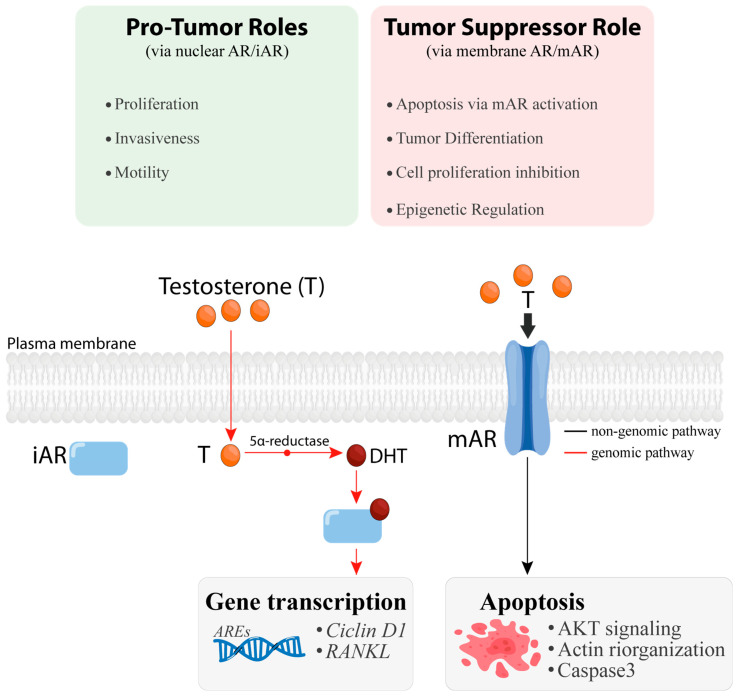
Androgen signaling in CRC. The pathway indicated by the red arrows represents the genomic signaling route, whereby dihydrotestosterone (DHT) and testosterone (T) bind to intracellular androgen receptors (iARs). Following ligand binding, the receptor complex translocates to the nucleus, where it interacts with specific DNA promoter regions known as androgen response elements (AREs). The pathway indicated by the black arrows represents the non-genomic signaling route. This is initiated by testosterone binding to membrane androgen receptors (mARs). Upon activation, these receptors trigger multiple downstream signaling pathways. The green box highlights the pro-tumorigenic effects of androgens in CRC, while the pink box shows their protective effects. Hsp, Heat Shock Protein; NF-kB, Nuclear factor kappa-light-chain-enhancer of activated B cells; COX-2, Cyclooxygenase-2; RANKL, Receptor Activator of Nuclear Factor kB ligand; MAPK, Mitogen-Activated Protein Kinase; ERK, Extracellular signal-Regulated Kinase; PI3K, Phosphoinositide 3-kinase; Protein Kinase B (AKT); JNK, c-Jun N-terminal Kinase. Adapted from citations [69,89].

Structurally, the AR consists of four functional domains: the N-terminal domain (NTD), responsible for transcriptional activation; the DNA-binding domain (DBD), which recognizes specific regulatory sequences; the hinge region, containing the nuclear localization signal; and the ligand-binding domain (LBD), which binds T and DHT and includes the AF2 domain required for coactivator recruitment [28]. Ligand binding induces dissociation of AR from heat shock protein 90 (HSP90), followed by receptor translocation to the nucleus and activation of androgen-responsive gene (ARE) transcription [89] (see Figure 5).

Functionally, AR activation promotes cell proliferation, survival, and systemic inflammation, whereas its inhibition reduces cell viability and enhances sensitivity to chemotherapeutic agents, highlighting the potential of anti-androgen strategies in CRC. The role of androgens in colorectal carcinogenesis is complex and remains incompletely understood, with inconsistent findings across experimental and clinical studies. Preclinical investigations by Abancens et al. demonstrated a potential tumor-promoting effect of androgens: castration reduced the number of colonic adenomas, while administration of T or other androgens increased tumor incidence. Paradoxically, prolonged androgen deprivation therapy has also been associated with an elevated risk of CRC, suggesting that hormonal balance rather than absolute androgen levels may influence tumor susceptibility [90,91,92].

Experimental models support this role: androgen deprivation decreases the incidence of intestinal tumors induced by carcinogens such as azoxymethane (AOM), while testosterone administration restores tumor susceptibility [93,94]. Similarly, T has been shown to stimulate proliferation of intestinal tumor cells, whereas its depletion suppresses this effect. Androgens also influence stromal signaling by modulating nerve growth factor (NGF) receptor pathways and increasing expression of bone morphogenetic protein (BMP) inhibitors, thereby promoting intestinal organoid growth and affecting proliferation and differentiation [89]. In AOM/DSS models, orchiectomy reduces both colitis severity and distal colon tumor incidence in males, whereas T administration upregulates inflammatory mediators such as COX-2 and iNOS, exacerbating inflammation and promoting progression to invasive carcinoma. Consistent findings have been reported in genetic models (ApcPirc/+ and ApcMin/+), where males develop more adenomas than females and androgen reduction decreases tumor burden [93,94,95].

Other preclinical studies demonstrate sex-specific differences, with males showing greater susceptibility to CRC development. Orchiectomy reduces tumor formation, while testosterone replacement increases tumor burden. In female-derived clinical samples, increased AR expression appears to promote a more aggressive phenotype through modulation of specific β-tubulin isoforms, a mechanism that may contribute to the higher mortality observed in men [71]. However, some studies report absent AR expression in colonic tissue and adenomas, suggesting that T may act predominantly through systemic mechanisms, such as modulation of inflammation, rather than direct epithelial signaling [7]. Clinical data are similarly inconclusive. Some studies report that higher circulating total T or sex hormone-binding globulin (SHBG) levels are associated with a reduced CRC risk, whereas elevated free T appears to correlate with increased risk. Notably, prostate cancer patients undergoing androgen deprivation therapy exhibit a higher incidence of CRC, particularly in the distal colon [7].

In female-derived clinical samples, increased AR expression appears to promote a more aggressive phenotype through modulation of specific β-tubulin isoforms, a mechanism that may contribute to the higher mortality observed in men [71]. Despite substantial evidence supporting a pro-tumorigenic role of androgens, other studies describe inverse associations. Higher levels of free T have been linked to reduced CRC incidence and mortality, and T replacement therapy has been associated with earlier-stage tumor diagnosis. Additionally, obesity, often accompanied by altered androgen levels, is a known risk factor for CRC in men [96]. Conversely, several studies have found no significant association between T, SHBG, or other androgens and the risk of precancerous lesions or CRC [7].

There are several factors that could explain the discrepancies between the pro-tumorigenic effects of androgens in preclinical models and the increased CRC risk associated with androgen deprivation or low T in humans. Firstly, the aromatization hypothesis suggests that androgens are essential for local estrogen production in the colon. Given the well-established tumor-suppressive role of the estrogen/ERβ axis, androgen depletion (whether pathological or iatrogenic via androgen deprivation therapy) may indirectly promote carcinogenesis by reducing the substrate available for local estrogen synthesis [7,71]. Secondly, the inverse association between T and CRC risk in clinical cohorts may be confounded by metabolic status. Low circulating T is a hallmark of obesity and metabolic syndrome in men, conditions characterized by chronic inflammation and increased CRC risk [2,96]. Finally, these paradoxical findings highlight the limitations of current experimental models. While acute androgen administration typically promotes proliferation in animal studies, human CRC often develops in a context of chronic hormonal decline or prolonged androgen deprivation therapy. This may activate distinct systemic inflammatory pathways or alter stromal-epithelial signaling in ways that are not fully replicable in preclinical settings [90].

Collectively, these findings suggest that the impact of androgens on colorectal carcinogenesis depends on a complex interplay between hormonal metabolism, systemic inflammation, and tumor biology. From a prognostic perspective, elevated AR expression has been associated with larger tumor size, poor differentiation, advanced stage, lymph node involvement, and metastasis, indicating an unfavorable prognosis [85,97]. Sex- and age-related differences have also been reported: in men, AR expression is higher in early-stage right-sided tumors, whereas in women it increases with age and is more pronounced in postmenopausal compared to premenopausal women, suggesting a role for circulating hormone levels in modulating receptor biology [9].

### 5.3. Progesterone

Progesterone (P4) is primarily produced by the ovaries, the placenta and the adrenal glands. It plays a central role in regulating the menstrual cycle, pregnancy, and numerous cellular processes, including proliferation, apoptosis, and differentiation. In CRC, however, the role of progesterone and its receptor (PR/PGR) is less well understood than that of estrogen, and experimental and clinical results are often inconclusive [7,85,98]. While some studies report low or absent PR expression in colorectal tumors and cell lines, suggesting limited involvement, others describe a progressive increase in receptor expression along the normal-adenoma–adenocarcinoma sequence. These studies hypothesize that PR plays a role in neoplastic progression [99,100,101]. Reduced P4 levels or poor PR expression have been associated with poorer prognoses and more aggressive phenotypes. Conversely, PR activation appears to limit proliferation and promote apoptosis, suggesting a potential modulatory and protective role.

**Figure 6:**
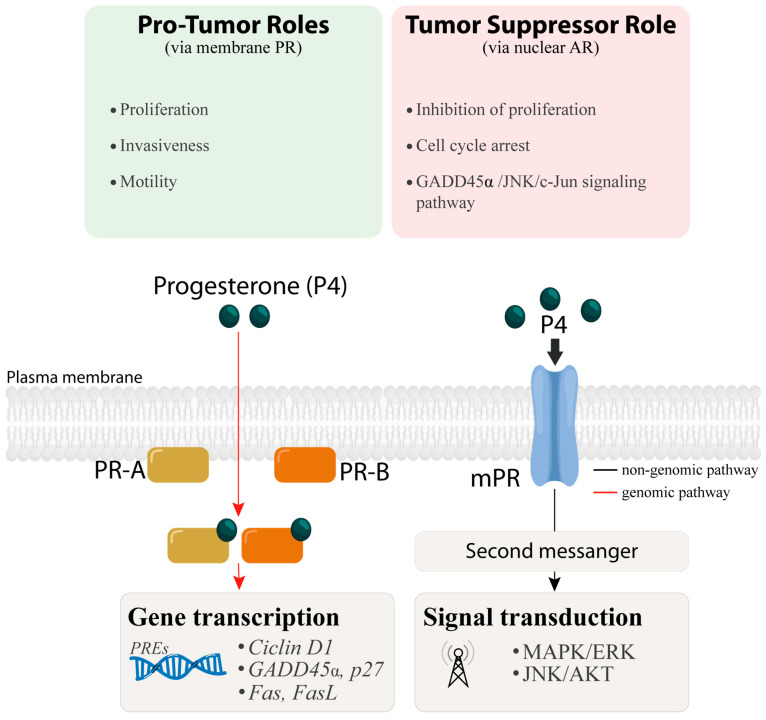
Mechanism of progesterone action in CRC. Progesterone (P4) exerts its effects by binding to progesterone receptors (PR-A and PR-B). After binding, the PRs translocate to the nucleus, where they bind to specific DNA promoter regions called progesterone response elements (PREs), thereby regulating several genes (the genomic pathway, red arrows). P4 also induces rapid, non-genomic effects (black arrows) mediated by membrane progesterone receptors (mPRs). The green box highlights progesterone’s pro-tumorigenic effects in CRC, while the pink box shows its protective effects. ERK1/2, Extracellular signal-Regulated Kinase; NF-kB, Nuclear factor kappa-light-chain-enhancer of activated B cells; RANKL, Receptor Activator of Nuclear Factor kB ligand; GADD45 α, Growth Arrest and DNA-Damage-inducible protein alpha; Fas, Fas cell surface death receptor; FasL, Fas Ligand; MAPK, Mitogen-Activated Protein Kinase; ERK, Extracellular signal-Regulated Kinase; PI3K, Phosphoinositide 3-kinase; AKT, Protein Kinase B; JNK, c-Jun N-terminal Kinase. Adapted from citations [69,89,98,101].

At the molecular level, P4 acts through genomic and non-genomic mechanisms. There are two main isoforms of nuclear PR: PR-A and PR-B. Both are characterized by an N-terminal domain, a DNA-binding domain (DBD), a hinge region and a ligand-binding domain (LBD). Binding P4 to the LBD induces a conformational change, resulting in dissociation from chaperone proteins, dimerization, and nuclear translocation. Once in the nucleus, PR binds to progesterone response elements (PREs), thereby regulating genes involved in proliferation, cycle arrest, and apoptosis, such as Cyclin D1, RANKL, GADD45α, p27, Fas, and FasL [98,101]. Activation of nuclear PR promotes cell cycle arrest and apoptosis by modulating the JNK/GADD45α pathway and inhibits oncogenic pathways such as the FRα/c-Src/ERK1/2/NF-κB/p53 pathway [89,101]. Meanwhile, P4 can activate non-genomic pathways via membrane receptors or membrane-associated PR isoforms. This induces rapid responses involving MAPK, ERK, JNK and Akt (see Figure 6). These signals regulate cell motility, stress response and proliferation, independently of gene transcription. They can also integrate with genomic signaling to create a coordinated network influencing growth, apoptosis, and cell differentiation [7,101].

Experimental studies also suggest that P4 may have a synergistic effect with estrogen. Combining E2 and P4 enhances the anti-tumor effects by jointly modulating ERβ and PR and counteracting ERα-mediated proliferative pathways [89]. Activation of PR is also necessary for the antiproliferative effects of certain agents, such as folate, highlighting the receptor’s role in regulating tumor growth [101]. PR expression in CRC varies and may depend on sex, age, and tumor location. Higher levels have been observed in premenopausal women, suggesting that PR may contribute to the lower incidence of CRC in young women [7].

Overall, P4 and PR emerge as complex regulators of colorectal carcinogenesis, integrating genomic and non-genomic signals to modulate proliferation, apoptosis and oncogenic pathways. Despite the heterogeneity of clinical data, experimental evidence supports a potential protective role for P4, particularly when used in conjunction with estrogens. This suggests that PR could be a promising target for preventing and personalizing the treatment of CRC [7,90].

## 6. Sex Differences in Gut Microbiota

Over the last decade, research in the fields of oncology and microbiology has fundamentally shifted our understanding of CRC pathogenesis by establishing the existence of substantial gender-specific disparities in the composition, diversity and function of the gut microbiome. Indeed, recent evidence from primary cohort studies, cross-sectional analyses and systematic reviews demonstrates that sexual dimorphism plays a critical, multifaceted role in modulating the interaction between the host and commensal or pathogenic microorganisms. These interactions significantly influence the onset and progression of malignancy through distinct hormonal, immune and metabolic pathways [90,102].

The human gut contains a complex ecosystem of approximately 40 trillion microorganisms, including archaea, viruses, fungi and bacteria. Bacteria play a key role in modulating the host’s metabolism by absorbing indigestible carbohydrates, producing vitamins B and K, and promoting, maturing and developing innate and cell-mediated immunity. They also contribute to maintaining intestinal barrier function and an adequate immune response against pathogens [103,104]. Under normal physiological conditions, intestinal bacteria and the host coexist in a state of homeostasis. Several studies implicate microbial dysbiosis, a pathological imbalance in the microbial community, as a critical driver of CRC [1,105,106], through mechanisms involving interaction with the immune system [107], induction of inflammation and oxidative stress [108], cross-talk with the endocrine system [7,18], and production of bioactive compounds, including metabolites, toxins, and virulence factors [109,110,111].

**Figure 7:**
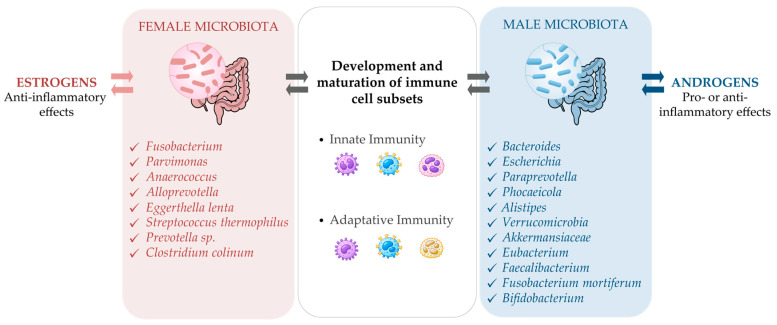
Sex differences in gut microbiota and their interaction with the immune and endocrine systems. Male and female individuals display distinct gut microbiota composition and diversity (“gut microgenderome”), which contribute to sex-specific differences in innate and adaptive immunity by influencing immune cell development. The microbiota and sex hormones interact bidirectionally (sex steroid–gut microbiome axis): estrogens tend to promote an anti-inflammatory microbial profile and maintain intestinal homeostasis, whereas androgens are often associated with gut dysbiosis and a more pro-inflammatory environment, contributing to the observed sexual dimorphism in CRC incidence and progression. In turn, gut bacteria can modulate sex hormone levels (e.g., via estrogen metabolism or androgen production), potentially impacting gonadal function (gut microbiota–gonadal axis). The figure shows the most common bacteria found in patients with CRC. Adapted from citations [105,115].

Recent high-throughput sequencing and meta-analyses studies have revealed that the composition and function of the gut microbiome are not uniform across sexes but instead exhibit significant sex-specific patterns (see Figure 7). The diversity and composition of gut microbes differ between males and females from birth, increasing during puberty and diminishing with age, particularly in postmenopausal women [112,113,114,115,116]. The literature suggests that, in healthy populations, women generally exhibit higher microbial diversity and richness than men, which is often characterized by a higher Firmicutes/Bacteroidetes ratio [7]. However, the stability of this diversity during CRC development varies drastically by sex. While alpha diversity (species richness) remains relatively stable in males throughout the progression from healthy tissue to carcinoma, it decreases significantly in females. Concurrently, beta diversity (community composition) patterns show opposing trends: CRC progression is associated with an increase in beta diversity in men, suggesting a divergence of microbial communities, whereas it is associated with a decrease in women, implying a convergence towards a specific dysbiotic profile. Therefore, the stability of this diversity during CRC development varies greatly depending on sex [117].

From a taxonomic perspective, a disparity in bacterial specificity has been observed between males and females in the onset of CRC. Male patients exhibit a significant increase in potentially genotoxic and pro-inflammatory bacteria. Studies utilizing shotgun metagenomic and third-generation sequencing have identified elevated abundances of *Bacteroides*, *Escherichia*, *Paraprevotella*, *Phocaeicola* and *Alistipes* in males. Furthermore, taxa such as *Verrucomicrobia*, *Akkermansiaceae*, *Eubacterium* and *Faecalibacterium* are frequently reported as being enriched in male CRC cohorts. Conversely, the microbiome of female CRC patients is characterized by an enrichment of immunosuppressive pathobionts. Prominent taxa identified in women include *Fusobacterium*, *Parvimonas*, *Anaerococcus*, *Alloprevotella*, *Eggerthella lenta* and *Streptococcus thermophilus*. Further findings suggest that, although men may harbor higher levels of *Fusobacterium mortiferum* and *Bifidobacterium adolescentis*, women exhibit specific increases in *Prevotella* sp. and *Clostridium colinum* [118,119]. It is important to note that there are some discrepancies in the literature, particularly about the genus *Bacteroides*. Depending on the stage of the disease and the species-specific resolution, some studies find it to be more prevalent in females, highlighting the complexity of these associations. These contradictory findings may be due to variations in stool processing techniques, differences in the sequencing technology employed (ribosomal 16S versus next-generation sequencing), subject age, diet and other environmental factors.

The taxonomic differences between males and females are underpinned by the complex interplay between sex hormones and the gut microbiota, which is often referred to as the ‘microgenderome’ [7]. The sex steroid-gut microbiome axis is all about how sex steroids and gut microbes work together, with the sex steroids affecting the gut microbiota and the microbiota changing the levels of sex steroids. A study conducted by Shin and colleagues investigated the link between gut microbes and T (in men) and E2 (in women). The study found that patients with high T or E2 levels had a more diverse gut microbiome [120]. Changes in the microbiota that occur at different life stages in association with hormonal shifts support the direct influence of sex steroids [7].

Estrogen and testosterone act as the main modulators of this axis. An animal study demonstrated how the gut microbiome affected intestinal metabolism and the deglucuronidation of androgens. Indeed, by measuring unconjugated and glucuronidated androgen levels, it was seen that, in young adult males, unconjugated DHT was 70-fold higher in the faeces than in the serum. The distal intestine of germ-free mice exhibited high levels of glucuronidated T and DHT, but very low levels of free DHT [121].

The ‘microgenderome’ determines distinct metabolic capacities, particularly with regard to the metabolism of sex steroid hormones, a concept collectively termed the ‘estrobolome’. The estrobolome is defined as the aggregate of enteric bacterial genes capable of metabolizing estrogens. Biochemically, endogenous estrogens (primarily E2 and estrone) undergo conjugation (glucuronidation and sulphation) in the liver to facilitate their excretion into the gastrointestinal tract via the bile. Once in the colon, specific bacterial species that possess β-glucuronidase enzymes catalyze the deconjugation of these inactive estrogen glucuronides. This enzymatic cleavage releases free, biologically active estrogens, which can then act locally on the colonic mucosa via ERβ or be reabsorbed into the portal circulation via enterohepatic circulation [122,123,124,125,126,127].

Key bacterial species driving this estrobolome activity include members of the *Bacteroidetes* and *Firmicutes phyla*, such as *Bacteroides uniformis*, *Bacteroides vulgatus*, *Clostridium perfringens*, *Streptococcus pasteurianus* and *Escherichia coli*. Sex-specific dysbiosis directly alters this enzymatic loop. For example, women with CRC often have fewer -glucuronidase-producing commensals, resulting in insufficient deconjugation and lower bioavailability of mucosal E2. This leads to a subsequent loss of protective ERβ-mediated anti-tumor signaling [125,126,127].

The interplay between microbiota and sexual hormones is fundamental to understanding the varying susceptibility to CRC between sexes.

Estrogens, particularly E2, are widely regarded as protective. It promotes beneficial microbiota and inhibits CRC cell proliferation through anti-inflammatory mechanisms and by enhancing DNA mismatch repair. It also inhibits tumor cell proliferation by activating ERβ [126,127]. Research suggests that women with CRC frequently have lower levels of microbial beta-glucuronidase activity, which, as previously mentioned, could reduce the bioavailability of circulating estrogens and diminish their protective effects [126]. Furthermore, estrogen supplementation in animal models has been shown to reverse dysbiosis by increasing microbial diversity and the commensal-to-opportunistic pathogen ratio, thereby reducing tumor burden. Specifically, E2 treatment in male mice was found to reduce the abundance of *Bacteroides* and decrease the *Firmicutes*/*Bacteroidetes* ratio, bringing the microbiome closer to a ‘female-like’, less tumor-promoting profile [7,128].

Unlike estrogen, androgens appear to promote colorectal tumor formation, which could explain the higher incidence of CRC in men. High T levels have been associated with specific changes in the gut microbiota, such as reduced diversity and increased opportunistic pathogens [127]. Indeed, the male microbiome, which is enriched with species such as *Escherichia coli*, *Alistipes finegoldii* and *Bacteroides fragilis*, interacts differently with the steroid pool. High androgen levels and distinct β-glucuronidase profiles further promote a pro-inflammatory microenvironment that is favorable to tumor formation. In male mouse models, T administration has been shown to exacerbate azoxymethane/dextran sulfate sodium-induced carcinogenesis, increasing the abundance of pathobionts such as *Akkermansia muciniphila*, while depleting the probiotic *Parabacteroides goldsteinii*. Conversely, orchiectomy has been shown to result in a microbial shift that mitigates tumor growth [7,129].

Therefore, the bidirectional interaction between host sex hormones and intestinal microbiota creates distinct physiological environments that modulate CRC risk differently in males and females [37,126,127]. Specifically, microbiota/sex hormone crosstalk has immunological implications and contributes to the sexual dimorphism observed in the tumor microenvironment. Female CRC patients typically present with an increase in tumor-infiltrating invariant natural killer T (iNKT) cells and a decrease in cytotoxic T lymphocytes (CTLs). Functional assays have demonstrated that the dysbiosis of gut microbiota found in female patients can actively impair the antitumor functions of these iNKT cells by interfering with the granzyme-perforin cytotoxic pathway [126]. Conversely, testosterone-driven pathways appear to trigger more pronounced inflammatory responses in males, whereby pathogenic bacteria induce gut barrier dysfunction and the recruitment of myeloid-derived suppressor cells (MDSCs) and M2-subtype tumor-associated macrophages (TAMs). This creates an immunosuppressive environment that facilitates tumor progression [127,129].

The functional output of the microbiota, particularly the metabolome, also exhibits sexual dimorphism. In male models of CRC, there is a significant upregulation of glycerophospholipid metabolism [129]. Specifically, lysophosphatidylcholine (LPC) has been identified as a male-specific oncometabolite that promotes cell proliferation and disrupts gut barrier function by downregulating tight junction proteins such as ZO-1 and Occludin. Conversely, metabolites such as L-arginine, which were depleted in males, have been shown to inhibit CRC cell growth [129].

Furthermore, due to lower levels of Lactobacilli and butyrate-producing bacteria than healthy women, healthy men have a higher risk of CRC due to the loss of the protective effects of short-chain fatty acids (SCFAs), such as butyrate. SCFAs are important metabolites resulting from the fermentation of food by gut microbes and are crucial for maintaining intestinal homeostasis [130]. Once produced in the colon, SCFAs are quickly absorbed by colonic cells and enter the citric acid cycle in mitochondria to generate ATP, thereby providing energy to the cells [122,123]. SCFAs also have anti-inflammatory properties and can prevent oxidative stress. They can improve intestinal barrier function and modulate immune responses, as well as exhibiting anti-CRC activity [124,131].

Taken together, these findings imply that sexual dimorphism in CRC is not solely a consequence of host genetics but rather is actively sustained and perpetuated by a sex-specific microbiota that interacts dynamically with the host’s hormonal environment.

## 7. Sex Differences in CRC Treatment

It is important to diagnose CRC as early as possible because it allows for identifying CRC when it is at a localized stage, reducing incidence and mortality and optimizing outcomes. In this case, the 5-year survival rate was supposed to be around 90%. Therefore, to identify CRC at an early stage developed countries introduced screening programs based on fecal occult blood tests or fecal immunochemical tests and colonoscopy. The choice of test can differ between countries. Literature evidence suggested that men and women differ from each other both in participation in screening programs and in the effectiveness of the screening test to detect CRC [132,133]. Indeed, females showed higher participation rates in screening programs [12]. The socioeconomic status, health behaviors, risk perception, access to care, education, sociocultural barriers, summarized as “gender-related factors”, were also reported to modulate screening participation. For instance, men exhibit lower risk perception in the absence of symptoms than women, which translates into skipping screening participation. On the other hand, the sensitivity of the screening tests seemed to be higher in men than in women who reported a better specificity. Women accounted for a higher rate of false negatives and lower rate of false positives, due to right-sided tumors, which bleed less, reducing test sensitivity and delaying CRC detection [12]. Consequently, women undergo further investigations with a lower frequency than men do, and then at diagnosis CRC is at a more advanced stage and is less differentiated; women are at an older age and present lymph node metastasis. Potential reasons responsible for the differences in the effectiveness of screening tests between sexes can be attributed to differential screening participation and levels of hemoglobin in stool, different tumor biology, sex hormones and tumor microenvironment. Regarding tumor biology, we can simplistically refer to two unique characteristics that characterize many neoplastic lesions in women. The first is the anatomical location, more common in the ascending colon, which, to a lesser extent than lesions in the descending colon, is associated with the presence of occult blood in the stool. The second is due to the different anatomy of the lesion, which, due to its flat configuration, can escape detection during colonoscopy and is also less prone to bleeding.

Therapeutic treatments for CRC can differ depending on the stage and site of the tumor and the physical condition of the patient. They usually include surgical intervention, radiotherapy, chemotherapy, targeted therapy, immunotherapy, and drug combination therapy. If the tumor is small and at a localized stage (i.e., stage I, II, III) surgery is recommended. Surgery is also required for tumors that show resistance to radiation and chemotherapy. Depending on the site where the tumor is found and its size, surgical procedure options for CRC can be represented by laparoscopy, radiofrequency ablation, cryoablation, or colostomy [134]. To reduce the risk of relapses, surgery is often followed by adjuvant chemotherapy with fluorouracil, leucovorin and oxaliplatin (FOLFOX regimens) or capecitabine plus oxaliplatin (CAPOX regimens) [135]. For stage IV CRC, which is considered not amenable to surgery, chemotherapy is the only choice of treatment [136].

### 7.1. Chemotherapeutic Agents

Chemotherapy is the major treatment option for CRC, either at the early stage and at the metastatic stage [137,138]. Alkylating agents, antimetabolites, plant alkaloids, and agents that influence biological responses are the main drugs utilized [139]. The backbone of CRC chemotherapy is represented by Fluoropyrimidine, in particular by 5-fluorouracil (5-FU), a synthetic fluorinated pyrimidine analog that acts by inhibiting the thymidylate synthetase enzyme, leading in turn to inhibition of DNA replication [138,140]. Literature data clearly highlight that sex has an influence on the pharmacokinetics of 5-FU [141,142]. Indeed, females can eliminate the drug to a lesser extent compared to males. Thus, females are exposed to the drug for a longer time than males and experience greater systemic exposure compared to males, caused by a reduced clearance of the drug [142,143]. Therefore, women reported higher grades of both toxicity and adverse events (i.e., alopecia, diarrhea, nausea and vomiting, anemia and neutropenia) than men did [144,145,146]. Despite higher toxicity, men and women display similar efficacy either in terms of overall survival or progression-free survival [145].

Different CRC therapeutic strategies are available nowadays, based on the combination of multiple drugs. For instance, leucovorin, a reduced folate able to stabilize fluorouracil’s interaction with the thymidylate synthetase enzyme, is administered with 5-FU [138,140]. Moreover, irinotecan, a natural alkaloid known as a Topoisomerase I inhibitor, and capecitabine, an oral prodrug of fluorouracil that undergoes a conversion to fluorouracil, are used in combination with 5-FU as well. For patients with metastatic CRC, capecitabine is used in combined therapy with irinotecan (XELIRI), with or without bevacizumab [147]. Interestingly, these combinations showed a trend similar to monotherapy. Indeed, they displayed sex-related differences regarding chemotherapy-related toxicity, as female patients experienced increased risk of adverse events and higher toxicity compared to males, without clear differences in progression-free and overall survival, overall response rate, or clinical benefit rate according to sex, supporting similar treatment efficacy between the two sexes [148,149,150]. A combination of fluoropyrimidines (e.g., 5-FU), oxaliplatin, and irinotecan is widely used. Regimens that combine 5-FU with oxaliplatin (OX) and capecitabine (CAP or XELODA or XEL) are used as well [151]. In the case of advanced CRC, treatments combining 5-FU and leucovorin with either oxaliplatin or irinotecan are the main and most widely used treatment options utilized. For instance, the FOLFIRI regimen, combining 5-FU with leucovorin and irinotecan, both very efficient in improving 5-FU efficacy, demonstrated a very strong ability to delay cancer progression, enhancing progression-free survival, overall survival and response rates compared to 5-FU alone [152,153]. Another chemotherapeutic protocol for CRC treatment, known as the FOLFOXIRI regimen, which combines irinotecan, oxaliplatin, and 5-FU/leucovorin, showed significant efficacy associated with well-tolerated side effects but increased compared to the FOLFIRI group. The third therapeutic option, the XELIRI regimen, based on the combination of irinotecan with capecitabine, demonstrated a profile comparable to FOLFIRI with the convenience of capecitabine’s oral administration but reported a higher gastrointestinal toxicity compared to FOLFIRI [154]. Several clinical studies have been conducted with the aim of exploring gender differences for patients with metastatic colorectal cancer. Some of them reported no differences in terms of overall survival, overall response rate, or clinical benefit rate according to sex. However, they showed a significantly higher toxicity in females, compared to males [149,150].

### 7.2. Targeted Therapies

Targeted therapies represent an effective alternative treatment option for CRC patients. It involves the use of small molecules, like monoclonal or therapeutic antibodies, that, once penetrating cancerous cells they bind to selected enzymes, inactivating them, leading in turn to inhibition of cancer cell growth and favoring apoptosis. Alternatively, they can bind specific receptors on the cell surface, regulating the pathways downstream of cell cycle progression and cell death directly. In addition, they can target immune cells, enforcing the immune system’s attack on cancer cells [151].

Regarding CRC treatments, inhibitors of vascular endothelial growth factor (VEGF) or epidermal growth factor receptor (EGFR) are currently used in combination with chemotherapy. Data reported that monoclonal antibodies against VEGF and EGFR can increase the overall survival of CRC to three years [155]. For instance, the humanized monoclonal antibody bevacizumab works by binding to VEGF, blocking in this way the formation of new blood vessels. It is usually administered in combination with fluorouracil and leucovorin or with FOLFIRI or FOLFOX in patients with metastatic colorectal cancer. Clinical data reported that bevacizumab was able to enhance the rate of response and progression-free survival (PFS) of CRC patients [154,156]. Frequent adverse effects were documented, such as mucositis, electrolyte imbalances, fatigue, anorexia, hypertension and rash. Interestingly, these studies showed that the overall survival is more or less the same between men and women, but females had benefits only once over 60 years and reported higher toxic effects at all ages compared to males [157,158].

Cetuximab and panitumumab are two monoclonal antibodies targeting EGFR effective in the treatment of metastatic CRC [159]. Cetuximab acts by binding to the external domain of EGFR, promoting internalization of the receptor and its degradation. It induces the blockage of survival pathways, which favors inhibition of cell growth, leading in turn to cell death induction and down-regulation of VEGF expression [160]. Panitumumab works similarly to cetuximab in metastatic CRC. Both antibodies demonstrated an effective prolongation of disease-free and overall survival when used in CRC patients already treated with chemotherapy or in case of chemotherapeutic treatment failure [151,153,161]. For instance, the Valentino study highlighted a similar outcome between men and women when panitumumab was added to the FOLFOX regimen [162]. Interestingly, the overall survival was slightly better in women than men, but men reported higher progression-free survival (PFS) [163] Data reported that patients with a higher number of EGFR copies and mutations in K-ras gene had a great benefit with EGFR inhibitors [153]. Unfortunately, a significant proportion of patients presenting K-ras mutations remain unresponsive to this therapy because they developed resistance to cetuximab or panitumumab treatments. In this case, alternative treatment utilizing small molecules able to inhibit specifically multiple tyrosine kinases, such as Regorafenib (Fluror-sorafenib, stivarga), is available [164].

CRC tumors that carry BRAF V600 mutations are more frequent in females who develop tumors with high MSI and prevalent right location [165]. Clinical studies assessed the efficacy of therapeutic treatments addressing specifically BRAF V600 mutations using inhibitors of BRAF, such as encorafenib. These studies reported that the combination of encorafenib plus cetuximab, with or without binimetinib, in the treatment of BRAF V600-mutated colorectal cancer patients is effective in both sexes, with significantly shorter PFS and OS in men as compared with women, but women experienced superior toxicity, mild-to-moderate in severity, especially with the triplet regimen. Diarrhea, nausea, vomiting, and acneiform dermatitis were the most common adverse events reported [166,167,168,169].

### 7.3. Immunotherapy

**Figure 8:**
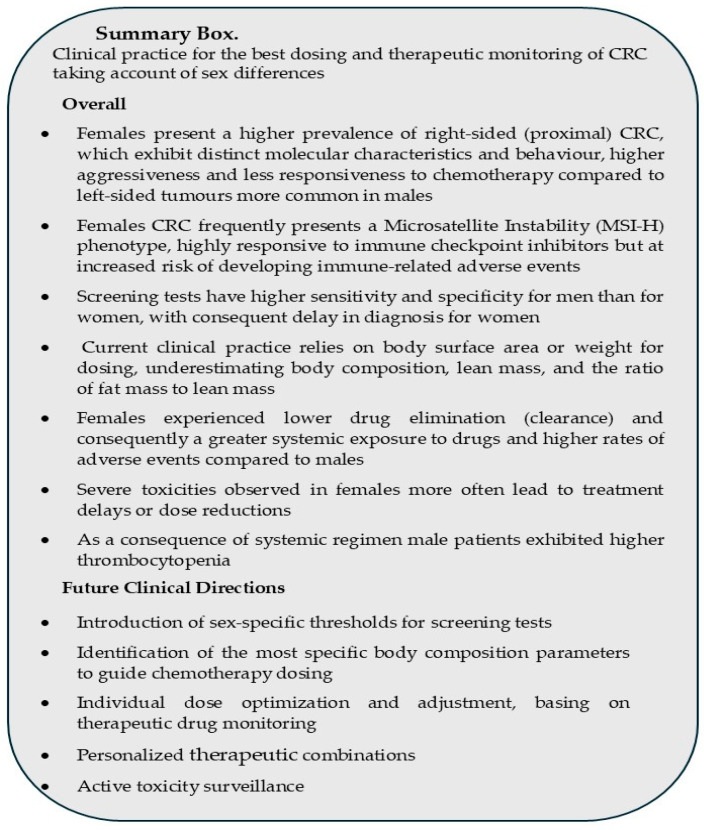
Summary box. The box summarizes the potential clinical practice for the optimization of dosing and monitoring of CRC accordingly to sex.

The fourth modality of treatment for metastatic CRC is immunotherapy, which utilizes specific immune checkpoint inhibitors (ICI) able to target specifically the PD-1/PD-L1 pathways [170]. This event favors the reactivation of the immune system’s response against the tumor [171]. To date, the best responses to immune checkpoint immunotherapies were recovered from metastatic CRC patients with deficiency in the mismatch repair system (dMMR) or with MSI-H, representing a small percentage of all CRCs of approximately 14% [172]. Indeed, studies reported that tumors with dMMR had a progression-free survival rate of approximately 78% and tumors with mismatch repair proficiency (pMMR) of approximately 11% [172]. Therefore, the use of anti-PD-1 mAbs, such as pembrolizumab and nivolumab, was authorized by the US Food and Drug Administration (FDA) for the treatment of solid tumors including CRC. Nowadays, immunotherapy represents the major treatment option for CRC patients presenting MSI or dMMR. Interestingly, males with mCRC treated with immune checkpoint inhibitors experienced better overall survival than females. [173]. Accordingly, additional data highlights a better response to ICI treatment and prolonged overall survival of men compared to women, due to higher DNA methylation levels associated with higher mutation counts [174]. Even if dMMR CRC or MSI-H CRC are more frequent in females, females generally experience higher adverse events and elevated toxicity. Females have stronger immune responses than men, which could be influenced by sex hormones, including estrogen, microbiota, and PDL-1 expression. Therefore, elevated immune response rates in females frequently generate auto-immunity phenomenon that in turn lead to higher adverse events. The data from these studies could also lead us to hypothesize that males might benefit from ICI monotherapy and from combination approaches targeting immune checkpoint inhibitors and methylation mechanisms. Whereas the best treatment option for females might be represented by a combination of ICI with IL-10 pathway inhibitors, or agents targeting myeloid-derived suppressor cells (MDSCs), or HLA-I expression enhancers [174]. In patients receiving immunotherapy the risk of objective non-hematologic toxicity was similar in both sexes, whereas the risk of symptomatic and hematologic adverse events (i.e., cardiovascular toxicity and bleeding) was higher for females compared with males [175] (Figure 8).

## 8. Conclusions

Colorectal cancer is a paradigmatic example of a malignancy in which biological sex acts as a pervasive and multidimensional determinant of risk, disease course, and therapeutic response. The evidence reviewed herein collectively demonstrates that the pronounced sexual dimorphism observed in CRC epidemiology, characterized by consistently higher incidence and mortality rates in men, a distinct anatomical predominance of right-sided tumors in women, and the so-called “female paradox” emerging after the sixth decade of life, is not merely a reflection of differential behavioral exposures, but is deeply rooted in divergent biological mechanisms operating at multiple levels. At the molecular level, cancer development differs between sexes. In women, tumors more frequently arise through microsatellite instability (MSI-H) and CpG island methylator phenotype (CIMP) pathways, whereas in men, the chromosomal instability (CIN) pathway is more prevalent. These differences are further influenced by sex chromosome–linked gene expression, distinct epigenetic regulation, and variations in the tumor microenvironment. Together, these factors highlight that sex-specific biological mechanisms play a significant role in cancer, emphasizing that precision oncology approaches should account for patient sex rather than applying uniform, sex-independent strategies.

Sex steroid hormones, particularly estrogens acting through ERβ, exert critical tumor-suppressive effects on colonic epithelium, and the progressive loss of ERβ expression during malignant transformation, compounded by postmenopausal hormonal decline, provides a mechanistic basis for the age-dependent convergence of CRC incidence between sexes. Androgens and progesterone further modulate this hormonal landscape through complex, context-dependent interactions with proliferative, apoptotic, and inflammatory pathways. Complementing the hormonal axis, the gut microbiome constitutes an additional sex-stratified layer of CRC susceptibility: the so-called “microgenderome”, shaped by the bidirectional interplay between sex steroids and microbial communities, contributes to distinct immunological and metabolic milieus that differentially modulate tumorigenesis in males and females. Finally, the documented sex-specific differences in the pharmacokinetics of chemotherapeutic agents, in the toxicity profiles of targeted therapies, and in the responsiveness to immune checkpoint inhibitors highlight that treatment stratification by sex is not merely advisable but clinically imperative.

Taken together, these findings advocate for the systematic integration of sex as a biological variable in the design of epidemiological studies, preclinical models, clinical trials, and screening protocols. Future research should prioritize the elucidation of the mechanistic crosstalk between sex hormones, gut microbiota, tumor immune microenvironment, and oncogenic signaling networks, with the ultimate aim of developing truly sex-informed preventive and therapeutic strategies for colorectal cancer.

In conclusion, it is crucial to address sex-specific disparities in CRC screening in order to reduce the burden of this disease. Future screening guidelines should move away from a ‘one-size-fits-all’ approach and adopt tailored strategies instead. Specifically, implementing gender-specific fecal hemoglobin (f-Hb) thresholds for FIT, such as lower cut-offs for women, could overcome the historically observed lower sensitivity in the female cohort. Furthermore, the age at which screening is initiated and its frequency must be optimized to account for the ‘female paradox’, ensuring that postmenopausal women, who are at a higher relative risk of presenting with aggressive right-sided lesions, are detected early. Integrating these sex-sensitive variables into routine clinical guidelines is a vital step towards achieving true precision oncology.

## Data Availability

No new data were created or analyzed in this study.

## Abbreviations

The following abbreviations are used in this manuscript:

AR	Androgen Receptor
CIN	Chromosomal Instability
CRC	Colorectal Cancer
DHT	Dihydrotestosterone
E2	17β-estradiol
EGFR	Epidermal Growth Factor Receptor
ER	Estrogen Receptor
GPER	G protein-coupled receptor
MSI	Microsatellite Instability
P4	Progesterone
PR	Progesterone Receptor
ROS	Reactive oxygen species
T	Testosterone
TME	Tumor Microenvironment
VEGF	Vascular Endothelial Growth Factor
5-FU	5-fluorouracil
